# Are Whiplash-Associated Disorders and Temporomandibular Disorders in a Trauma Related Cause and Effect Relationship? A Review

**DOI:** 10.3390/medicina59081482

**Published:** 2023-08-17

**Authors:** Nicola Montemurro, Irma Trilli, Ioana Roxana Bordea, Elisabetta Ferrara, Maurizio De Francesco, Francesca Caccamo, Giuseppina Malcangi, Biagio Rapone

**Affiliations:** 1 Department of Neurosurgery, Azienda Ospedaliero Universitaria Pisana (AOUP), University of Pisa, 56100 Pisa, Italy; nicola.montemurro@unipi.it; 2 Interdisciplinary Department of Medicine, “Aldo Moro” University of Bari, 70121 Bari, Italy; trilliirma@gmail.com (I.T.); giuseppinamalcangi@libero.it (G.M.); biagiorapone79@gmail.com (B.R.); 3 Department of Oral Rehabilitation, Faculty of Dental Medicine, University of Medicine and Pharmacy ‘Iuliu Hatieganu’, 400012 Cluj-Napoca, Romania; 4 Department of Medical, Oral and Biotechnological Sciences, University G. d’Annunzio, 66100 Chieti, Italy; igieneeprevenzione@gmail.com; 5 Department of Neurosciences, Institute of Clinical Dentistry, University of Padua, 35128 Padua, Italy; maurizio.defrancesco@unipd.it; 6 Benjamin Franklin Institute, 70032 Bitonto, Italy; caccamo.f78@libero.it

**Keywords:** whiplash, temporomandibular disorder, traumatic brain injury, temporomandibular joint, TMDs, TMJ, cervical spine

## Abstract

*Background*: Whiplash is associated with a wide variety of clinical manifestations, including headache, neck pain, cervical rigidity, shoulder and back pain, paresthesia, vertigo, and temporomandibular disorders (TMDs). Previous studies reported that TMDs are more common in individuals with chronic whiplash-associated disorders (WAD) than in the general population; however, the pathophysiology and mechanism of this relationship are still not well understood. *Methods*: A PubMed and Ovid EMBASE review was performed to identify all studies addressing the trauma related cause and effect relationship between WAD and TMDs from January 2003 to March 2023. *Results*: After screening for eligibility and inclusion criteria, a total of 16 articles met the selection criteria. The various included studies discussed different aspects of the association between WDA and TMDs, including changes in the coordination and amplitude of jaw opening, the severity of the associated symptoms/signs in cases of WAD, the degree of fatigue and psychological stress, difficulty in feeding, cervical and myofascial pain, changes in the MRI signal at various muscle points, muscle tenderness, and quality of life. *Conclusions*: In this review, we summarized the clinical evidence of any trauma related cause and effect relationship between whiplash and TMDs. An accurate screening of the previous literature showed that, in conclusion, the relationship between whiplash and TMDs is still unclear.

## 1. Introduction

Temporomandibular disorders (TMDs) refer to a group of conditions involving the orofacial region, divided into those affecting the masticatory muscles and those affecting the temporomandibular joint (TMJ) [1]. Whiplash-associated disorders (WADs) are the clinical manifestation of cranio-cervical injuries resulting from an acceleration–deceleration mechanism of energy transfer to the neck [2]. WAD may appear after a variable timeframe post-trauma. Due to the wide range of possible interactions between various signs and symptoms, the clinical scenario is exceedingly unpredictable. Whiplash is associated with a wide variety of clinical manifestations, including headache, neck pain, cervical rigidity, shoulder and back pain, para-aesthesia, vertigo, neck stiffness, problems with psychological distress, and TMDs [3]. Previous studies [3,4] reported that TMDs are more common in individuals with chronic WADs than in the general population; however, the pathophysiology and mechanism of this relationship are still not well understood. Over the years, some research has been carried out to understand if there is a, and what can be the, relationship between WADs and TMDs.

WAD is the term given to the collection of symptoms affecting the neck that are triggered by an accident with an acceleration–deceleration mechanism such as a motor vehicle accident [5]. The incidence of whiplash injury varies greatly between different parts of the world with significant monetary burden on the individual as well as the wider community. The cumulative incidence of patients seeking healthcare for whiplash arising from a road traffic accident has increased during the last 30 years to recent estimates of >3/1000 inhabitants in North America and Western Europe [6]. In addition, whiplash may indirectly damage the temporomandibular joint. Such injuries may affect the jaw’s normal function because the neck injury is linked to a disturbed control of mandibular and head–neck movements during jaw opening–closing tasks [7,8]. Additionally, pre-programmed orders and neural networks are shared by jaw and head and neck movements [9]. A whiplash injury damages the disc by shear stresses and the deep tissue of the facet joint by compression and/or stretching. Under normal conditions, the head is free to rotate, and no twisting forces are transmitted to the axial and subaxial structures [10]. The forces transmitted to the vertebrae, including the weight force and inertial forces, are not constant but variable over time [11]. The combined shear, flexion, and compression stresses in posterior hits might also harm the cervical facet capsular ligaments. Particularly vulnerable to damage are the cervical facet’s capsular ligaments, which can suffer damage at stress circumstances akin to those produced by whiplash [12]. Ginszt and colleagues [13] showed a relationship between the occurrence of myofascial trigger points (TrPs) within the upper trapezius and changes in the resting activity of the masticatory muscles. However, the relation mentioned above may only slightly concern the functional activity of the stomatognathic system. In numerous studies, disturbances in the masticatory symmetry were connected to pain in the head area and other symptoms related to TMDs and masticatory muscle pain [14,15]. Moreover, active myofascial TrPs in the cervical musculature may be responsible for headache reproduction in women with migraines [16].

To date, almost all hypotheses regarding the mechanism of rearend-impact-induced injury are based on kinematics [17]. It seems that the muscular trauma resulting from the impact acceleration is often explained in terms of neck hyperextension and hyperflexion [18], that can lead to affect the masticatory muscles or joints causing TMDs. In particular, the lateral pterygoid muscle can be injured in this way, leading to internal derangement and TMDs, which in turn influence rotation and translation of the disk and condyle [19]. Nonetheless, recent biomechanical investigations and MRI studies have not been able to explain fully the trauma related cause-and-effect-relationship TMD symptoms associated with disk-related injury [18]. In addition, several mechanisms could explain why women frequently have TMDs or WADs symptoms. Sensitivity to pain might be increased by hormonal changes that occur during a woman’s menstrual cycle or life cycle [20]. The musculature-related disparities between the sexes could be another mechanism, as human studies showed that men have larger muscle fibers and more type II fibers than women [21]; in addition, joint mobility is greater in women than in men [22].

Male and female muscle activity is affected by hormones [22]. The activity of red and white muscle fibers is both influenced by testosterone (present in 10% in women compared with the amount produced in men), while estrogen seems to act as a receptor on the ligaments influencing the tensor capacity, the load-bearing capacity of the joint, as well as ligamentous laxity in a transient manner [23,24]. In addition, hormonal changes are associated with the formation of muscle trigger points in muscles. They can cause transferred pain symptoms, causing aggravation alongside the formation of TMDs. Fernández-de-Las-Peñas et al. [25] showed the existence of multiple active muscle TrPs in the masticatory and neck–shoulder muscles in women with myofascial TMD pain and that referred pain areas were larger in TMD pain patients than in healthy controls. These results are in accordance with the notion of peripheral and central sensitization mechanisms in patients with myofascial TMDs [25].

Whiplash is considered by scholars to be a traumatic event that adversely affects the patient’s posture, which not only alternately and antithetically displaces the cranio-cervical and trunk structures damaging the osteo-arthro-muscular district, but also results in a disordered displacement of abdominal body fluids [26]. The body center of gravity would be altered creating compensatory muscle activities. This results in altered body posture with subsequent postural instability. Dental clenching, a condition of energy expenditure, is a phenomenon that provides postural balance in cases of a patient who has suffered whiplash or other trauma that has destabilized posture [27]. Both the temporomandibular joint and all structures of the stomatognathic system (dental arches and periodontium) are therefore subjected to pressure stress, causing joint pain and muscle hypertonus [28,29,30]. This is because body posture is managed by the unconscious subcortical component of the central nervous system (CNS), which processes proprioceptive and exteroceptive inputs into output, stimulating neuromotors and muscle activity (hypertone) [31]. During sleep, the CNS perceives the absence of postural stability references (visual and plantar support) and, in case of altered center of gravity, it activates the postural stability compensation systems with tightening and general muscle hypertonus [32,33].

The aim of this review is to investigate if there are any trauma related cause and effect relationship between WADs and TMDs to add a new contribution to the literature.

## 2. Materials and Methods

A PubMed and Ovid EMBASE review was performed to identify all studies addressing the trauma related cause and effect relationship of whiplash following cervical spine trauma with a temporomandibular joint clinical disorder. The following search terms were used from January 2003 to March 2023: whiplash OR whiplash injury AND temporomandibular disorder OR TMD OR TMJ OR temporomandibular joint disorders (Table 1). Before the start of the search, a review protocol was entered into the PROSPERO (or other) database (ID records n° 454427).

A total of 56 articles, including those listed in the references of the retrieved studies, were found originally. We then excluded the following items: all publications not dealing with whiplash and TMDs; all studies different from original articles (case report/case series, review, letters, commentaries, technical note, editorial); all preclinical studies; non-English written papers; and any other publication that did not comply with the goal of the present review. 

## 3. Results

**Figure 1:**
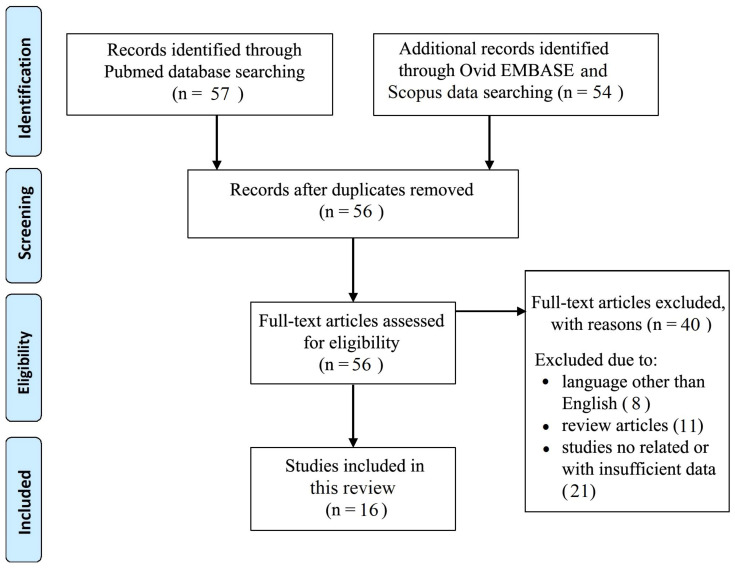
PRISMA flow diagram used in this literature review.

A total of 111 records were initially identified in the literature search, using Pubmed, Ovid, EMBASE, and Scopus databases, of which 55 were duplicates. After screening for eligibility and inclusion criteria, a total of 16 articles met the selection criteria [3,8,34,35,36,37] (Figure 1). The study and clinical characteristics are summarized in Table 2. 

The majority of publications were case–control studies (n = 11), followed by comparative studies (n = 3), observational prospective studies (n = 1), and retrospectives studies (n = 1). All studies included were rated as level 4 or 5 evidence for clinical research as detailed in the Oxford Centre for Evidence-Based Medicine 2011 guidelines. A total of 2779 patients with or without WAD and TMDs were included. The various included studies discussed different aspects of the association between WDA and TMDs, including changes in the coordination and amplitude of jaw opening [34], the severity of the associated symptoms/signs in cases of WAD [8,40], the degree of fatigue and psychological stress [35,36], difficulty in feeding [37], myofascial pain [39,41,42,45], changes in the MRI signal at various muscle points [43], muscle tenderness [44,46], and quality of life [36,39].

## 4. Discussion

The research diagnostic criteria for TMDs were introduced by Dworkin and LeResche in 1992 [48], who provided detailed standard instructions and identified three different subtypes, which are TMDs (join pain, arthralgia, and joint disorders), masticatory muscle disorders (muscle pain, contracture, and movement disorders), and headache [48,49]. 

### 4.1. Clinically Relevant Studies and Comparison with Previous Studies

One of the first studies that investigated the trauma related cause and effect relationship between whiplash and TMDs investigated in a cohort of 25 individuals with WAD the presence of any limitations in jaw function and found a decreased amplitude and a change in temporal coordination between the movements of the mandible and the head–neck complex [34]. Joint neck–jaw dysfunction is linked to neck trauma, and whiplash injuries may have a particular impact on jaw function. Following neck injuries, jaw functions, gaping, biting, chewing, swallowing, yawning, and communicating may be compromised [18,50,51]. During mandibular function, neural networks that coordinate mandibular and head and neck movements also engage cervical spine segments and extend caudally into the brainstem [52]. The central commands must be shared in the creation of these jaw networks and neck muscular synergies. Therefore, we suggest that future studies on the primary mechanism governing jaw function should focus on the role of cervical spine and head–neck motor control [53,54]. Generally, manual therapy, including TMJ mobilization and the soft tissue technique [55], improves TMJ function and reduces pain when applied to the cervical spine. This procedure alleviates pain via the neurological mechanisms responsible for reducing muscle activity, which may be due to the neuroanatomical connection and biomechanical relationship between these two components of the trigeminocervical complex [56]. In addition, previous studies demonstrated that the application of manual therapy or mobilization of the cervical spine could positively affect pain intensity in patients with TMD [57]. During the same years (2004), Klobas et al. [8] conducted a comparative case–control study between 54 patients with WAD and 66 patients without a history of WAD, showing that 89% of the WAD patients had severe symptoms and signs of TMDs compared with 18% of the control group, as well as a reduced mouth opening (48 mm vs. 54 mm) and increased pain on palpation of the jaw muscles and lateral palpation of the temporomandibular joint. In addition, Klobas et al. [8] reported that pain during jaw movement was present in 30% of the WAD group compared with 3% of the controls. Similar data were reported by Häggman-Henrikson and colleagues [35], who reported that 50 patients with WAD (compared with 50 patients with TMDs and 50 healthy subjects) had more difficulty, fatigue, and pain during unilateral chewing of a gum performed for 5 min. Visscher et al. [36] came to rather similar conclusions by studying three groups of patients (patients with chronic WAD pain, patients with chronic pain without WAD, and a group without neck pain) and described that the WAD group suffered more often from TMD pain and, more generally, from widespread pain than the group without neck pain and, finally, WAD patients showed higher degrees of psychological distress than the other two groups. Other studies also reported similar data [50,51]. A recent review showed the relationship between TMDs and bruxism and prosthodontics, showing that patients with TMDs may adapt less easily than healthy patients to the occlusal and psychological stress of a modification to their occlusal scheme because of their delicate psychophysiological equilibrium [58]. In routine clinical practice, the existence of clicking disc displacement sounds does not represent a contraindication to occlusal rehabilitations, whereas in patients with open-ended TMDs, their symptoms should be treated before beginning any prosthetic treatment [58]. Patients affected by TMDs are very sensitive to stressors and may thus adapt less easily than healthy patients to the occlusal and psychological stress of a modification to their occlusal scheme because of their delicate psychophysiological equilibrium [58].

Häggman-Henrikson et al. [41] conducted a case–control study that enrolled 70 individuals (40 women and 30 men) who had gone to the emergency room with neck pain following a car accident and 70 individuals (42 women and 28 men) who did not want to undergo a clinical examination but merely filled out evaluation questionnaires. These two case groups were compared with a matched control group of 70 individuals (42 women and 28 men) with no history of neck injury. Statistical investigations reported that WAD individuals had greater pain and dysfunction of the jaw than controls without any injury, as well as a higher likelihood of disability.

Similar results were presented by Salé et al. [40] who conducted a 15-year prospective study of 60 patients who were enrolled after whiplash and who were evaluated at the beginning, after a 1-year follow-up, and after 15 years of follow up and compared with a control group of 50 participants without a positive medical history for WDA, and who also found that whiplash injury symptoms were common both in the immediate and prolonged periods. Marini et al. [39] showed that the frequency of TMDs was higher following a whiplash injury, with a focus on myofascial pain and disc displacement with reduction. Salé et al. [38] warned physicians and health professionals when assessing symptoms related to a previous WDA, as their sample of 60 consecutive patients analyzed at time 0 and after 1 year of therapy showed that a certain percentage (40%) had inaccurate memories, with possible errors in recall, additions, and/or omissions. 

Furthermore, Grönqvist et al. [37] showed that patients with WDA were more likely to suffer from eating problems including biting, chewing, swallowing, and yawning, and part of the sample under investigation reported avoiding hard food and large pieces, and taking breaks during meals. Lampa E. et al. [42] conducted a case–control study involving 80 patients examined within 1 month after a WDA comparing them with 80 controls without neck trauma. Participants who had experienced a WDA had a higher Jaw Disability Checklist (JDC) together with higher Neck Disability Index (NDI) values and greater fatigue and pain during the chewing test.

Lee and colleagues [43] conducted a retrospective–transverse study of 100 patients (50 women, 50 men, mean age: 37.60 years) who underwent detailed evaluations for trauma history and clinical and MRI findings. Women perceived more pain and presented more tenderness on palpation than men, with also higher volume and signal changes in the lateral pterygoid muscle and greater anterior disc dislocation without reduction. Concerning the possibility of developing TMDs as a result of WDA, Sharma et al. [45] described that subjects with a higher sensitivity to heat pain were more prone to develop painful TMDs than subjects with a lower sensitivity to heat, and these data contributed to understanding the complex landscape around this topic, as highlighted by Almoznino et al. [44] who found a positive correlation between cervical tenderness (CTS) and history of WDA.

Bal et al. [46] conducted a retrospective study of 237 patients with TMDs to assess the relationships between direct trauma and whiplash injuries, but found no statistically significant difference based on gender, frequency of diagnosis, clip, crepitus, and trauma; furthermore, a history of direct trauma and WDA was described in 18.6% and 14.8% of the patients, and no relationship between clips, crepitus, and trauma was recorded.

Corsalini and colleagues [3] described a single-institution clinical case history of 31 patients following whiplash injury of which 20 patients (age 20–39 years) were treated with Zimmer collars and 11 patients (age 20–39 years) without collar treatment. In the study group, at 6-month follow-up, 3/5 patients showed a VAS score of 3, 4, and 5, while the remaining 2/5 patients reported no symptoms. In the control group, 4/11 patients needed an occlusal brace with a VAS of 2. This study showed that acute WADs are often self-limiting and, when they become chronic, are mostly present in patients with severe injuries, with previous TMDs, and later in life.

In addition to analyzing the relationships between WDA, TMDs, and the possibility of involvement of the auditory system [59,60,61,62], Lee et al. [47] investigated 90 patients (64 women, 26 men, mean age: 39.36 +/− 15.40 years) with 45 patients suffering from TMDs after WDA and 45 patients matched in age and gender with idiopathic causes of TMDs. These results showed that the pain reported by the VAS scale and the palpation index of the neck were significantly higher in the TMD group, with pain in the masseter, temporalis, lateral pterygoid, and medial muscles. In addition, structural changes in the masticatory muscles were predominantly evident in the TMDs. These data constituted a pathophysiological basis of different damage between the two groups, with a worse outcome in the TMDs. Klobas et al. [63] attempted to examine the impact of a particular therapeutic jaw exercise in order to determine whether and to what extent improvements were possible. During the years 2001–2002, ninety-four consecutive WAD patients who had been referred to and accepted for a course of therapy at a functional evaluation and rehabilitation clinic were enrolled by the authors. The patients adhered to a regimen of pain management, occupational therapy, and physical therapy. They were evaluated by a rehabilitation medicine specialist at the outset of their stay, as well as by a dentist who conducted a functional evaluation of the stomatognathic apparatus. In accordance with the inclusion criteria, 55 of the 93 patients who agreed to participate in the study had TMDs and chronic WADs. They were randomly divided into a control group (n = 30) and a group that underwent therapeutic jaw exercises (n = 25). Except for an increase in the maximum active mouth-opening capacity in the control group, the authors identified no inter- or intra-group differences in the symptoms and indications of TMDs at baseline, nor at 3-week and 6-month follow up. This specific therapeutic jaw exercise program, as a single rehabilitation modality, was not additional to the physical therapy, the occupational therapy, and pain management in reducing symptoms and signs of temporomandibular disorders in patients with chronic whiplash-associated disorders [60].

Another alternative capable of assisting the management of WAD symptoms, particularly headaches, has proven to be the administration of Botulinum Toxin (TB) [64,65]. In particular, two papers tried to address this possibility by screening two cohorts of patients suffering from such symptoms following whiplash trauma. The paper by Freund and Schwartz [66] studied patients who had been administered TB and who did not show significant improvement in their symptoms, until there was a reduction in the average intensity of pain four weeks after injection. In another paper [67], a cohort of patients was studied of whom 22 (representing 71%) showed some improvement after TB treatment. Although low-quality evidence indicates that digital health interventions can improve quality of life and overall treatment in the reduction of musculoskeletal pain, it is possible that the use of telehealth will improve the lives of these patients in the future [68,69].

### 4.2. Suggestions for Future Studies

Prospective research on whiplash populations should give a comparison with controls and provide a complete picture of the key factors impacting TMDs. The use of MRI, joint blocks, or injections, standardized, valid, and reliable clinical examinations, and appropriate outcome measurements could be used to identify these variables. Other findings that have been linked to whiplash and TMDs in studies should be considered as potential variables or descriptors to be defined in whiplash and TMDs populations. Examples include balance and postural abnormalities, shoulder–neck headaches, stress and psychosocial factors, parafunctional and adaptive habits, centrally mediated pain factors, and others [70,71,72].

### 4.3. Limitations

Although this article used general inclusion/exclusion criteria, it lacked important criteria with a flowchart demonstrating the steps of exclusion based on the identification of studies included with specific research designs such as randomized control trials/case-control studies/cohort studies and identification of studies with specific statistical approaches and sample size. Other limitations include the lack of quality scoring and formal blinded appraisal of studies reviewed. 

## 5. Conclusions

In this review, we summarized the clinical evidence of any trauma related cause and effect relationship between whiplash and TMDs. An accurate screening of the previous literature showed that a true relationship between WADs and TMDs is still unclear. Although an association is most likely, it is unknown whether its strength is clinically significant. Further studies are needed to elucidate more the mechanisms of this relationship in order to improve the clinical and surgical treatment of these diseases.

## Figures and Tables

**Table 1:** Search syntax.

PubMed Search Accessed between January 2003 and March 2023 (57 Articles)	Embase Search Accessed between January 2003 and March 2023 (54 Articles)
(whiplash OR whiplash injury) AND (temporomandibular disorder OR TMD OR TMJ OR temporomandibular joint disorders)	(‘whiplash’ OR ‘whiplash injury’) AND (‘temporomandibular disorder’ OR ‘TMD’ OR ‘TMJ’ OR ‘temporomandibular joint disorders’)

**Table 2:** Papers included in this literature review.

Authors	Year	Study Population	Results
Eriksson et al. [34]	2004	Patients (25)	Reduction of width and change in coordination
Klobas et al. [8]	2004	Patients with WAD (54); patients without WAD (66)	89% severe signs/symptoms (WAD); 18% sign/symptoms (without WAD)
Häggman-Henrikson et al. [35]	2004	Patients with WAD (50); patients with TMDs (50); controls (50)	WAD patients show more fatigue and pain than TMDs and control patients
Visscher et al. [36]	2005	Patient with pain and WAD; patient with pain, without WAD; patient without neck pain	WAD patients have more widespread pain and psychological distress than other groups
Grönqvist et al. [37]	2008	Patient with WAD pain (50); patient without WAD pain (50)	Relationship between WAD patients and impaired jaw function and eating disorders
Salé et al. [38]	2010	Patients with previous WAD following for 1 year (40)	High degree of inaccurate memories among within 1 year of WAD trauma
Marini et al. [39]	2013	Patients with WAD and oro-facial pain (65); patients with oro-facial pain without WAD (65)	Patient with WAD show increased frequency of myofascial pain and disc displacement with reduction
Salé et al. [40]	2014	Patients with WAD (60); patients without WAD (50)	More signs/symptoms in patients with WAD
Häggman-Henrikson et al. [41]	2016	Patients with WAD (70); patients with neck pain (70); controls (70)	WAD patients show more disability and pain than other groups
Lampa et al. [42]	2017	Patients WAD (80); patients without WAD (80)	WAD patients have disability and pain measured by JDC and other Parameters
Lee et al. [43]	2019	Man with oro-facial pain and WAD (50); women with oro-facial pain and WAD (50)	Women show more pain, tenderness on palpation, signal change in the lateral pterygoid muscle and more anterior disc displacement without reduction
Almoznino et al. [44]	2019	Patients with TMDs (192); control group (99)	Muscle tenderness scores were positively associated with TMDs disease characteristics and concurrent pain conditions
Sharma et al. [45]	2020	Patients with TMDs (233); control group (176)	Jaw injury was strongly associated with an elevated risk of painful TMDs
Bal et al. [46]	2020	Patients with TMDs (237)	Direct trauma and/or whiplash in patients with TMDs were not associated with diagnostic recordings and TMDs sounds
Lee et al. [47]	2021	Patients with TMDs and WAD (45); patients with TMDs without WAD (45)	VAS, PI, and PI of the neck indices were significantly higher in the with TMDs group than in the group with TMD but no WDA.
Corsalini et al. [3]	2022	patients with WAD treated with Zimmer collars (31); patients with WAD not treated (11)	TMDs are often self-limiting within a few months. The pre-existing TMDs and the age of the patient appears to be independent of the use of the Zimmer collar.

WAD, whiplash-associated disorder; TMDs, temporomandibular disorders; JDC, Jaw Disability Checklist; VAS, visual analog scale; PI, palpation index.

## References

[1] Maini K., Dua A. (2023). Temporomandibular Syndrome. [Updated 2023 Jan 30]. StatPearls [Internet].

[2] Spitzer W.O., Skovron M.L., Salmi L.R., Cassidy J.D., Duranceau J., Suissa S., Zeiss E. (1995). Scientific monograph of the Quebec Task Force on Whiplash-Associated Disorders: Redefining “whiplash” and its management. Spine.

[3] Corsalini M., Capodiferro S., Dell’olio F., Albanese G., Quaranta N., Solarino B., Catapano S., Di Venere D. (2022). Cranio-Mandibular Disorders after Whiplash Injury: A Mono-Institutional Clinical Study on 31 Patients. Int. J. Environ. Res. Public Health.

[4] Yaklai S., Chaweewannakorn C., Sastravaha P. (2023). A Nonsurgical Treatment Strategy for Longstanding, Nonreducible, Bilateral Temporomandibular Joint Dislocation: A Case Report. J. Oral Maxillofac. Surg..

[5] Pastakia K., Kumar S. (2011). Acute whiplash associated disorders (WAD). Open Access Emerg. Med..

[6] Ioppolo F., Rizzo R.S., Alpini D., Brugnoni G., Cesarani A. (2014). Epidemiology of Whiplash-Associated Disorders. Whiplash Injuries.

[7] Mishra R., Deora H., Florez-Perdomo W.A., Moscote-Salazar L.R., Garcia-Ballestas E., Rahman M.M., Shrivastava A., Raj S., Chavda V., Montemurro N. (2022). Clinical and Radiological Characteristics for Recurrence of Chronic Subdural Hematoma: A Systematic Review and Meta-Analysis. Neurol Int..

[8] Klobas L., Tegelberg A., Axelsson S. (2004). Symptoms and signs of temporomandibular disorders in individuals with chronic whiplash-associated disorders. Swed. Dent. J..

[9] Eriksson P.-O., Häggman-Henrikson B., Zafar H. (2007). Jaw–neck dysfunction in whiplash-associated disorders. Arch. Oral Biol..

[10] Montemurro N., Cocciaro A., Liberti G., Cosottini M., Perrini P. (2022). The internal trabecular bone structure of the odontoid process of the axis. A retrospective single-center comparative study in patients following cervical trauma. J. Neurol. Surg. Part A Central Eur. Neurosurg..

[11] Montemurro N., Perrini P., Mangini V., Galli M., Papini A. (2019). The Y-shaped trabecular bone structure in the odontoid process of the axis: A CT scan study in 54 healthy subjects and biomechanical considerations. J. Neurosurg. Spine.

[12] Siegmund G.P., Myers B.S., Davis M.B., Bohnet H.F., Winkelstein B.A. (2000). Human Cervical Motion Segment Flexibility and Facet Capsular Ligament Strain under Combined Posterior Shear, Extension and Axial Compression. Stapp Car Crash J..

[13] Ginszt M., Szkutnik J., Zieliński G., Bakalczuk M., Stodółkiewicz M., Litko-Rola M., Ginszt A., Rahnama M., Majcher P. (2022). Cervical Myofascial Pain Is As-sociated with an Imbalance of Masticatory Muscle Activity. Int. J. Environ. Res. Public Health.

[14] Mapelli A., Zanandréa Machado B.C., Giglio L.D., Sforza C., De Felício C.M. (2016). Reorganization of muscle activity in patients with chronic temporomandibular disorders. Arch. Oral Biol..

[15] Santana-Mora U., Cudeiro J., Mora-Bermúdez M., Rilo-Pousa B., Ferreira-Pinho J., Otero-Cepeda J., Santana-Penín U. (2009). Changes in EMG activity during clenching in chronic pain patients with unilateral temporomandibular disorders. J. Electromyogr. Kinesiol..

[16] Florencio L.L., Ferracini G.N., Chaves T.C., Palacios-Ceña M., Ordás-Bandera C., Speciali J.G., Falla D., Grossi D.B., Fernández-De-Las-Peñas C. (2017). Active Trigger Points in the Cervical Musculature Determine the Altered Activation of Superficial Neck and Extensor Muscles in Women With Migraine. Clin. J. Pain.

[17] Yoganandan N., Pintar F.A., Gennarelli T.A. (2002). Biomechanical Mechanisms of Whiplash Injury. Traffic Inj. Prev..

[18] Lee Y.-H., Lee K.M., Auh Q.-S., Hong J.-P. (2018). Magnetic Resonance Imaging-Based Prediction of the Relationship between Whiplash Injury and Temporomandibular Disorders. Front. Neurol..

[19] Wang M.Q., Yan C.Y., Yuan Y.P. (2001). Is the superior belly of the lateral pterygoid primarily a stabilizer? An EMG study. J. Oral Rehabil..

[20] Ribeiro-Dasilva M.C., Fillingim R.B., Wallet S.M. (2017). Estrogen-induced monocytic response correlates with TMD pain: A case control study. J. Dent. Res..

[21] Toft I., Lindal S., Bønaa K.H., Jenssen T. (2003). Quantitative measurement of muscle fiber composition in a normal population. Muscle Nerve.

[22] Carmichael M.A., Thomson R.L., Moran L.J., Wycherley T.P. (2021). The Impact of Menstrual Cycle Phase on Athletes’ Performance: A Narrative Review. Int. J. Environ. Res. Public Health.

[23] Shahraki S.F., Minoonejad H., Tabrizi Y.M. (2020). Comparison of some intrinsic risk factors of shoulder injury in three phases of menstrual cycle in collegiate female athletes. Phys. Ther. Sport.

[24] Pallavi L.C., Souza U.J.D., Shivaprakash G. (2017). Assessment of Musculoskeletal Strength and Levels of Fatigue during Different Phases of Menstrual Cycle in Young Adults. J. Clin. Diagn. Res..

[25] Fernández-de-Las-Peñas C., Galán-Del-Río F., Alonso-Blanco C., Jiménez-García R., Arendt-Nielsen L., Svensson P. (2010). Re-ferred pain from muscle trigger points in the masticatory and neck-shoulder musculature in women with temporomandibular disoders. J. Pain.

[26] Rodriquez A.A., Barr K.P., Burns S.P. (2004). Whiplash: Pathophysiology, diagnosis, treatment, and prognosis. Muscle Nerve.

[27] Fujino S., Takahashi T., Ueno T. (2010). Influence of voluntary teeth clenching on the stabilization of postural stance disturbed by electrical stimulation of unilateral lower limb. Gait Posture.

[28] Majcen Rosker Z., Kristjansson E., Vodicar M. (2023). How well can we detect cervical driven sensorimotor dysfunction in concus-sion patients? An observational study comparing patients with idiopathic neck pain, whiplash associated disorders and con-cussion. Gait Posture.

[29] Puerta de Diego R., Elia Martinez J.M., Gallart Úbeda V., Meliá Casado B., Tenias Burillo J.M. (2021). Alteraciones posturográficas y oculomotoras en las primeras 24 horas tras un latigazo cervical [Posturographic and oculomotor findings in the first 24 hours after whiplash]. Ehabilitación.

[30] Juul-Kristensen B., Clausen B., Ris I., Jensen R., Steffensen R., Chreiteh S., Jørgensen M., Søgaard K. (2013). Increased neck muscle activity and impaired balance among females with whiplash-related chronic neck pain: A cross-sectional study. J. Rehabil. Med..

[31] De Pauw R., Coppieters I., Kregel J., De Meulemeester K., Danneels L., Cagnie B. (2016). Does muscle morphology change in chronic neck pain pa-tients?—A systematic review. Man. Ther..

[32] O’Shaughnessy T. (1994). Craniomandibular/temporomandibular/cervical implications of a forced hyper-extension/hyper-flexion episode (i.e., whiplash). Funct. Orthod..

[33] Miles T.S. (2007). Postural control of the human mandible. Arch. Oral Biol..

[34] Eriksson P.-O., Zafar H., Haggman-Henrikson B. (2004). Deranged jaw-neck motor control in whiplash-associated disorders. Eur. J. Oral Sci..

[35] Häggman-Henrikson B., Österlund C., Eriksson P.-O. (2004). Endurance during chewing in whiplash-associated disorders and TMD. J. Dent. Res..

[36] Visscher C., Hofman N., Mes C., Lousberg R., Naeije M. (2005). Is temporomandibular pain in chronic whiplash-associated dis-orders part of a more widespread pain syndrome?. Clin. J. Pain.

[37] Grönqvist J., Häggman-Henrikson B., Eriksson P.-O. (2008). Impaired jaw function and eating difficulties in whiplash-associated disorders. Swed. Dent. J..

[38] Salé H., Hedman L., Isberg A. (2010). Accuracy of patients’ recall of temporomandibular joint pain and dysfunction after experi-encing whiplash trauma: A prospective study. J. Am. Dent. Assoc..

[39] Marini I., Paduano S., Bartolucci M.L., Bortolotti F., Bonetti G.A. (2013). The prevalence of temporomandibular disorders in patients with late whiplash syndrome who experience orofacial pain: A case-control series study. J. Am. Dent. Assoc..

[40] Salé H., Bryndahl F., Isberg A. (2014). A 15-year follow-up of temporomandibular joint symptoms and magnetic resonance imaging findings in whiplash patients: A prospective, controlled study. Oral Surg. Oral Med. Oral Pathol. Oral Radiol..

[41] Häggman-Henrikson B., Lampa E., Marklund S., Wänman A. (2016). Pain and Disability in the Jaw and Neck Region following Whiplash Trauma. J. Dent. Res..

[42] Lampa E., Wänman A., Nordh E., Häggman-Henrikson B. (2017). Effects on jaw function shortly after whiplash trauma. J. Oral Rehabil..

[43] Lee Y., Lee K.M., Auh Q., Hong J. (2019). Sex-related differences in symptoms of temporomandibular disorders and structural changes in the lateral pterygoid muscle after whiplash injury. J. Oral Rehabil..

[44] Almoznino G., Zini A., Zakuto A., Zlutzky H., Bekker S., Shay B., Haviv Y., Sharav Y., Benoliel R. (2019). Muscle tenderness score in temporo-mandibular disorders patients: A case-control study. J. Oral. Rehabil..

[45] Sharma S., Ohrbach R., Fillingim R.B., Greenspan J.D., Slade G. (2020). Pain Sensitivity Modifies Risk of Injury-Related Tem-poromandibular Disorder. J. Dent. Res..

[46] Bal B., Koksal T., Ebeoglu B., Oral K. (2020). Retrospective analysis of trauma incidence in patients with temporomandibular dis-orders. Dent. Traumatol..

[47] Lee Y.-H., Lee K.M., Auh Q.-S. (2021). MRI-Based Assessment of Masticatory Muscle Changes in TMD Patients after Whiplash Injury. J. Clin. Med..

[48] Dworkin S.F., LeResche L. (1992). Research diagnostic criteria for temporomandibular disorders: Review, criteria, examinations and specifications, critique. J. Craniomandib. Disord..

[49] Scarola R., Montemurro N., Ferrara E., Corsalini M., Converti I., Rapone B. (2021). Temporomandibular Disorders and Fibromyalgia: A Narrative Review. Open Access Maced. J. Med. Sci..

[50] Carroll L.J., Ferrari R., Cassidy J.D. (2007). Reduced or painful jaw movement after collision-related injuries: A population-based study. J. Am. Dent. Assoc..

[51] Noreña A.J., Fournier P., Londero A., Ponsot D., Charpentier N. (2018). An Integrative Model Accounting for the Symptom Cluster Triggered After an Acoustic Shock. Trends Hear..

[52] Perrini P., Montemurro N. (2016). Congenital absence of a cervical spine pedicle. Neurol. India.

[53] Karamian B.A., Schroeder G.D., Lambrechts M.J., Canseco J.A., Oner C., Vialle E., Rajasekaran S., Dvorak M.R., Benneker L.M., Kandziora F. (2022). An international validation of the AO spine subaxial injury classification system. Eur. Spine J..

[54] Lambrechts M.J., Schroeder G.D., Karamian B.A., Canseco J.A., Oner F.C., Benneker L.M., Bransford R.J., Kandziora F., Rajasekaran S., El-Sharkawi M. (2023). Effect of surgical experience and spine subspecialty on the reliability of the AO Spine Upper Cervical Injury Classification System. J. Neurosurg. Spine.

[55] Lee I.-S., Kim S.-Y. (2023). Effectiveness of manual therapy and cervical spine stretching exercises on pain and disability in myofascial temporomandibular disorders accompanied by headaches: A single-center cohort study. BMC Sports Sci. Med. Rehabil..

[56] Bartsch T., Goadsby P.J. (2003). Increased responses in trigeminocervical nociceptive neurons to cervical input after stimulation of the dura mater. Brain.

[57] Suvinen T.I., Hanes K.R., Reade P.C. (1997). Outcome of therapy in the conservative management of temporomandibular pain dysfunction disorder. J. Oral. Rehabil..

[58] Minervini G., Fiorillo L., Russo D., Lanza A., D’amico C., Cervino G., Meto A., Di Francesco F. (2022). Prosthodontic Treatment in Patients with Temporomandibular Disorders and Orofacial Pain and/or Bruxism: A Review of the Literature. Prosthesis.

[59] Haggman-Henrikson B., Zafar H., Eriksson P.O. (2002). Disturbed jaw behavior in whiplash-associated disorders during rhythmic jaw movements. J. Dent. Res..

[60] Iglebekk W., Tjell C., Borenstein P. (2015). Treatment of chronic canalithiasis can be beneficial for patients with vertigo/dizziness and chronic musculoskeletal pain, including whiplash related pain. Scand. J. Pain.

[61] Rapone B., Inchingolo A.D., Trasarti S., Ferrara E., Mancini A., Montemurro N., Scarano A., Inchingolo A.M., Dipalma G., Inchingolo F. (2022). Long-Term Outcomes of Implants Placed in Maxillary Sinus Floor Augmentation with Porous Fluorohydroxyapatite (Algipore^®^ FRIOS^®^) in Comparison with Anorganic Bovine Bone (Bio-Oss^®^) and Platelet Rich Plasma (PRP): A Retrospective Study. J. Clin. Med..

[62] Vielsmeier V., Strutz J., Kleinjung T., Schecklmann M., Kreuzer P.M., Landgrebe M., Langguth B. (2012). Temporomandibular Joint Disorder Complaints in Tinnitus: Further Hints for a Putative Tinnitus Subtype. PLoS ONE.

[63] Klobas L., Axelsson S., Tegelberg Å. (2006). Effect of therapeutic jaw exercise on temporomandibular disorders in individuals with chronic whiplash-associated disorders. Acta Odontol. Scand..

[64] Linde M., Hagen K., Stovner L.J. (2011). Botulinum toxin treatment of secondary headaches and cranial neuralgias: A review of evidence. Acta Neurol. Scand..

[65] Epstein J.B., Klasser G.D. (2011). Whiplash-associated disorders and temporomandibular symptoms following motor-vehicle colli-sions. Quintessence Int..

[66] Freund B.J., Schwartz M. (2000). Treatment of Chronic Cervical-Associated Headache With Botulinum Toxin A: A Pilot Study. Headache.

[67] Freund B.J., Schwartz M. (2002). Use of Botulinum Toxin in Chronic Whiplash-Associated Disorder. Clin. J. Pain.

[68] Valentijn P.P., Tymchenko L., Jacobson T., Kromann J., Biermann C.W., AlMoslemany M.A., Arends R.Y. (2022). Digital Health Interventions for Musculoskeletal Pain Conditions: Systematic Review and Meta-analysis of Randomized Controlled Trials. J. Med. Internet Res..

[69] Montemurro N. (2022). Telemedicine: Could it represent a new problem for spine surgeons to solve?. Global Spine J..

[70] Fernandez C.E., Amiri A., Jaime J., Delaney P. (2009). The relationship of whiplash injury and temporomandibular disorders: A narrative literature review. J. Chiropr. Med..

[71] Moss R., Lombardo T., Villarosa G., Simkin L., Hodgson J. (1995). Oral habits and TMJ dysfunction in facial pain and non-pain subjects. J. Oral Rehabil..

[72] Rubin A.M., Woolley S.M., Dailey V.M., A Goebel J. (1995). Postural stability following mild head or whiplash injuries. Am. J. Otol..

